# Autonomous Sequence Generation for a Neural Dynamic Robot: Scene Perception, Serial Order, and Object-Oriented Movement

**DOI:** 10.3389/fnbot.2019.00095

**Published:** 2019-11-15

**Authors:** Jan Tekülve, Adrien Fois, Yulia Sandamirskaya, Gregor Schöner

**Affiliations:** ^1^Institute for Neural Computation, Ruhr-University Bochum, Bochum, Germany; ^2^Lorraine Research Laboratory in Computer Science and its Applications, Vandœuvre-lès-Nancy, France; ^3^Institute for Neuroinformatics, University of Zürich and ETZ Zürich, Zurich, Switzerland

**Keywords:** neural dynamic modeling, autonomous robot, sequence generation, scene perception, reaching movement

## Abstract

Neurally inspired robotics already has a long history that includes reactive systems emulating reflexes, neural oscillators to generate movement patterns, and neural networks as trainable filters for high-dimensional sensory information. Neural inspiration has been less successful at the level of cognition. Decision-making, planning, building and using memories, for instance, are more often addressed in terms of computational algorithms than through neural process models. To move neural process models beyond reactive behavior toward cognition, the capacity to autonomously generate sequences of processing steps is critical. We review a potential solution to this problem that is based on strongly recurrent neural networks described as neural dynamic systems. Their stable states perform elementary motor or cognitive functions while coupled to sensory inputs. The state of the neural dynamics transitions to a new motor or cognitive function when a previously stable neural state becomes unstable. Only when a neural robotic system is capable of acting autonomously does it become a useful to a human user. We demonstrate how a neural dynamic architecture that supports autonomous sequence generation can engage in such interaction. A human user presents colored objects to the robot in a particular order, thus defining a serial order of color concepts. The user then exposes the system to a visual scene that contains the colored objects in a new spatial arrangement. The robot autonomously builds a scene representation by sequentially bringing objects into the attentional foreground. Scene memory updates if the scene changes. The robot performs visual search and then reaches for the objects in the instructed serial order. In doing so, the robot generalizes across time and space, is capable of waiting when an element is missing, and updates its action plans online when the scene changes. The entire flow of behavior emerges from a time-continuous neural dynamics without any controlling or supervisory algorithm.

## 1. Introduction

Neurally inspired robotics already has a long history. To position our work in this history and review our conceptual commitments, we discuss three strands of neurallly inspired robotics.

### 1.1. Reactive Behaviors

One strand goes back to Grey's electronic turtle (Grey, 1950) and Braitenberg's thought experiments on vehicles (Braitenberg, 1984). This line of work reached maturity in behavior-based robotics (Brooks, 1991; Mataric, 1998) in which flexibility emerges from the coordination of elementary behaviors, each establishing a direct link from sensory inputs to actuators, in the manner of reflex loops. This is particularly suited to conceptual “vehicles,” robotic systems in which the sensors are mounted on the moving actuator. This enables closed loop situations that greatly reduce the demands on representation and abstraction. For instance, a visual sensor mounted in a robot hand makes it possible to achieve reaching by visual servoing without an explicit representation of objects in the world (Ruf and Horaud, 1999).

By organizing closed action-perception loops in architectures, most famously the subsumption architecture (Brooks, 1986), this form of reactive robotics may generate behaviors of a certain complexity (Proetzsch et al., 2010). The behavior is generated *autonomously* in the sense that sensory information from a structured environment may trigger the activation of elementary behaviors, which may lead to chains of activation and deactivation events through the architecture, inducing sequences of behavioral decisions, without the need for an explicit internal plan, schedule, or program. The organization of such behaviors is implicitly encoded in the architecture itself.

Avoiding representation and abstraction is a feature of the approach (Brooks, 1990), but also points to a limitation of this line of neurally inspired robotics: Behavior-based robots are not very good at cognition. Minimally, cognition is engaged when the link between sensing and acting becomes less direct. Building and exploiting memory is an example (Engels and Schöner, 1995). So when an action is based on sensory information that is no longer directly available on the sensory surface at the time the action unfolds, relevant information must be represented in memory. Memories are useful only if they are represented in a form in which they remain invariant under changes the system experiences between the acquisition of the memory and its use. For instance, the memory representation of a movement target for a vehicle needs to be invariant under rotation of the vehicle (Bicho et al., 2000). A more demanding form of cognition is the capacity to perceive sequences of events and store them in a memory for serial order so that a sequence with a matching serial order can then be acted out (such as hearing a phone number and then dialing it). Again, the information needs to abstract from the sensor data to be useful for the required actions.

Our approach is historically based on behavior-based thinking, which we extended by adding neural memory representations and neural mechanism of decision making (Schöner et al., 1995). Here we will study how memory for serial order can be built and used to act sequentially in new environments.

### 1.2. Neuronal Oscillators and Pattern Generators

A second strand of neurally inspired robotics is based on the idea that neural oscillators may generate rhythmic movement patterns. That idea has been used to generate legged locomotion in biologically inspired robots (Holmes et al., 2006; Ijspeert, 2008). Such neural oscillator ideas can be integrated with the dynamics of limbs and muscles and their interaction with the ground, enabling stable locmotion patterns (Full and Koditschek, 1999; Ghigliazza et al., 2003). Neural oscillators are one important class of neural networks in which recurrent connections are strong enough to induce endogenous patterns of neural activation that are not mere transformations of input. That class can be extended to neural timers that generate complex temporal patterns that may be the basis for certain motor skills (Buonomano and Laje, 2010). Coupling neural oscillators provides an account for coordination (Schöner and Kelso, 1988) and adaptation enables the modulation of rhythmic movement patterns (Aoi et al., 2017).

Typically, however, these kinds of models do not address how movement may be directed at targets in the world, such as when reaching for an object or intercepting a ball. A related class of neural models going back, perhaps, to Bullock and Grossberg (1988), generates time courses by integrating neural activity toward an end-point that may ultimately be determined by perceptual processes. This is the basis of the notion of dynamic movement primitives (Schaal et al., 2003), which is still broadly neurally inspired although it is typically implemented in a mathematical form that does not explicitly reference neural processing principles (see Ijspeert et al., 2013 for an excellent review). The dynamical systems framework for reaching toward objects can address how such movement is directed at objects in the world (Hersch and Billard, 2008). Typically, however, the representation of the object's pose and kinematic state remains clearly outside the neural metaphor (while achieving superhuman performance in skills such as catching, Kim et al., 2014).

Our approach builds on this tradition of using neural oscillators for timing. We generate individual goal-directed reaches from an active transient solution of a recurrent neural dynamics. We extend this tradition by providing a neural dynamic architecture that obtains from the visual array a neural representation of the targets of a reaching movement. This requires that an object's visual coordinates are transformed into coordinates anchored in the initial position of the hand (Schöner et al., 2019). We show how such a neural representation of movement targets may be linked to the visual array, enabling online updating of movement generation when the scene changes (see Knips et al., 2017 for an earlier version of such online updating).

### 1.3. Neural Networks for Perception

A third strand of neural inspiration for embodied cognitive systems is, of course, the use of neural networks to extract relevant information about the environment from sensory (e.g., image, sound) data (Kriegeskorte, 2015). This strand is currently undergoing explosive growth as the scaling of deep neural networks in size and learning examples enables superhuman performance in certain classification and detection tasks (Lecun et al., 2015; Schmidhuber, 2015). These neural networks essentially serve as intelligent filters of sensory information, a critical function when robot cognition is to be linked to the world.

While these networks by themselves do not perform cognitive functions, they may provide outputs that enable cognition. For instance, networks may deliver labels for a relational description of a visual scene (e.g., Kelleher and Dobnik, 2017). In most cases, the actual reasoning about spatial or other relations is, however, performed outside a neural processes model, based on algorithms and probabilistic inference. First steps are being made, however, toward such models generating the sequential attentional selection on which human visual cognition is centrally based (Ba et al., 2015).

Our approach is based on the classical notion of feature extraction along the visual pathway, the simplest step in these kinds of systems (e.g., Serre et al., 2007). As we do not address object recognition, we limit ourselves to very simple features here (see Lomp et al., 2017 for how the approach may link to object recognition). Instead, we demonstrate how a neural dynamic system may autonomously generate the sequence of attentional selections to build a visual scene memory that is intermittently coupled to the visual array, and thus is sensitive to change and capable of updating in response to such change.

### 1.4. Goals

In this paper, we integrate these three strands of neurally inspired robotics which requires us to extend each of them. Our emphasis is on how the integrated system—essentially a network of neural dynamic populations—is continuously or intermittently coupled to sensory information, while at the same time being capable of autonomously generating sequences of decisions, actions, and events. Neural activation is thus generated endogenously is this system, while retaining the coupling to the sensory surfaces.

The system addressed four key elements of grounded cognition: (1) It autonomously builds scene memory, a neural map of locations and feature values bound to those locations. Different objects are sequentially brought into the attentional foreground, in each case creating an entry into scene memory, which can be updated if change is detected. (2) The system learns the serial order of events that occur in its visual array. Each time an attended object changes, the system registers the transition and learns the new feature value as associated with its serial position. This provides a possible interface through which a human user can interact with the system. (3) The system generates a sequence of actions oriented at objects in the world in the learned serial order. At any point in the sequence, this entails finding an object in the visual surrounding that matches the feature values currently sought, generating the action, and then transitioning to the next sub-task within the learned sequence. This exemplifies the capacity of the system to autonomously generate organized behavior that is not merely reactive but reflective of a learned plan. (4) Each action consists of a pointing gesture oriented at an attended object. The action is initiated once the object has been brought into the attentional foreground, but may be updated any time if object shifts to a new location. This is a minimal instantiation of object-oriented action that any form of cognitive robotics must be capable of.

Key to this demonstration is the notion of neural dynamics, in which strongly recurrent neural networks, approximated as spatially and temporally continuous neural fields, evolve primarily under the influence of their internal interaction that sets up attractor states. Inputs induce instabilities that bring about switches of neural states from which sequences of cognitive or motor states emerge. Such neural dynamics are capable of making decisions, building working memories, and organizing sequential transitions (Schöner et al., 2016). Because their neural states are stable, neural fields retain their functional properties when they are coupled to other fields. Fields may thus serve as building blocks of networks of fields, which could be thought of as neural dynamic architectures. These networks may be coupled to sensory inputs, while evolving under their own, endogenous dynamics, resolving the tension between reactive and cognitive systems.

To make the ideas accessible, we restrict the demonstration to a very simple scenario. A robot observes a table top on which a human user places and removes colored objects in a particular serial order. The user then builds a new visual scene, that includes the objects with colors contained in the taught series. The robot points at these objects in the order defined by the human teacher. When an object of the next required color is not available, the system waits until such a color is presented. When the visual array changes, the robot updates its reaching plans. This may happen online if the change occurs while the robot is already attempting to point at the object. All action and observation run autonomously in neural dynamics. There is no control algorithm outside the neural dynamics. See Supplementary Videos 1 and 2 for exemplary demonstrations of teaching and executing the series.

## 2. Dynamic Field Theory

We use Dynamic Field Theory (DFT) (Schöner et al., 2016) as a conceptual framework. DFT provides neural process accounts for elementary cognitive functions such as decision making, memory creation, or the generation of sequences. The core elements of DFT are neural populations which may generate activation patterns that are not primarily dictated by input. This is based on structured and strong recurrent connectivity within the population. Excitatory recurrent connectivity enables detection decisions in which neural activation is induced by input, but then stabilized against decay even as input may weaken again. The initial detection occurs through an instability, in which the resting state becomes unstable. The detection is reversed when the activated state becomes unstable, typically at a lower level of input than needed for initial detection. If the excitatory recurrency is sufficiently strong, the reverse detection instability does not happen, leading to activation that is sustained even when the inducing input is removed entirely. This is the basis for working memory.

Inhibitory recurrent connectivity enables selection decisions, in which one sub-population becomes activated even if multiple sub-populations receive supra-threshold input. Such selection decisions are also stabilized so that the selection of a sub-population may persist even as inputs to other sub-populations become stronger (up to a limit, when the selection instability is encountered). So even though neural populations may be driven by input, they may realize non-unique mappings from input to activation states based on their activation history.

When different populations are coupled, they may induce these kinds of instabilities in each other. This is the basis for generating sequences of neural activation states. When the coupling occurs between excitatory and inhibitory sub-populations, the instabilities may trigger active transients, well-defined time courses of neural activation from temporally unstructured input. Neural oscillations are another possible dynamic regime.

Through their connectivity to sensory or motor surfaces, neural populations may effectively represent continuous feature dimensions, ***x***. This leads to the notion of neural dynamic fields, *u*(***x***). We employ a particular mathematical formalization of the dynamics of such neural populations that goes back to Amari (1977),

(1) $$\tau \dot{u}(x)=-u(x)+h+s+\int \sigma (u(x^{\prime }))\omega (x-x^{\prime })dx^{\prime },$$

where τ describes the field's relaxation time, *h* < 0 the field's resting level, *s* the sum of input stimuli, and ω the field's interaction kernel that defines the pattern of recurrent connectivity within the field. Only sufficiently activated field locations contribute to interaction or project onto other fields, as formalized by the sigmoidal non-linearity, σ(*u*). Thus, one may think of the activation variable, *u*, as something like a population-level membrane potential that reflects how close neurons in the population are to the firing threshold [other formalizations use the firing rate as a population variable, see Wilson and Cowan (1973)]. In the meantime, there is a large literature on the mathematics of such fields (Coombes et al., 2014).

**Figure 1:**
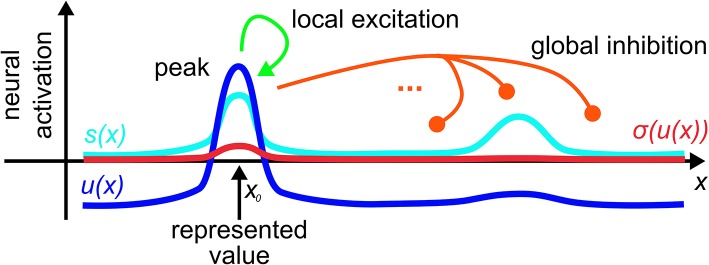
A dynamic neural field spanning a metric dimension, *x*, represents a specific value, *x*_0_, along that dimension through a supra-threshold activation peak that is stabilized by local excitatory and global inhibitory interactions.

The kernel, ω, combines short-range excitatory coupling with long-range inhibitory coupling. This leads to localized peaks of activation as the activation states that emerge from the instability of the resting state when localized input reaches a threshold (Figure 1). These peaks are the units of representation in DFT that specify through their locations particular values along the represented dimension.

Fields may represent low-dimensional metric spaces. When their dimensionality grows, the binding problem arises and can be solved, see Chapter 5 of Schöner et al. (2016). A limit case are zero-dimensional fields which can be thought of as populations of neurons that represent categorical states. These may arise from larger populations through inhomogoneities in the input or output connectivity. We sometimes call such zero-dimensional fields neural dynamic nodes and model them by single activation variables, *u*(*t*), subject to a neural dynamics analogous to Equation (1).

When fields of different dimensionality are coupled, new functions emerge (Zibner et al., 2011, see also Chapter 9 of Schöner et al., 2016). In projecting from a higher to a lower dimensional field, certain dimensions may be marginalized, which effectively probes for the existence of a peak anywhere along the marginalized dimensions. In projecting from a lower to a higher dimensional field, a boost may be given to a subspace, enabling locations within the subspace to reach the detection instability. This is the basis for visual search. The control of peak formation in a field through homogeneous boosting of its activation level is a mechanism of control that may effectively gate particular projections by enabling or disabling peak formation. This mechanism is also central to sequence generation through the condition of satisfaction (CoS) (Sandamirskaya and Schöner, 2010) that will play a central role in our sequence representation model. The neural representation of the CoS is a neural field or a neural node that is pre-activated by the currently active behavior. That behavior predicts the sensory or internal state that will indicate its successful completion. When a signal matching that prediction is received from sensory inputs or from other neural processes, the CoS goes through a detection instability. It then inhibits the current behavior in a reverse detection instability and enables the activation of a new behavior (Sandamirskaya and Schöner, 2010).

## 3. Model

**Figure 2:**
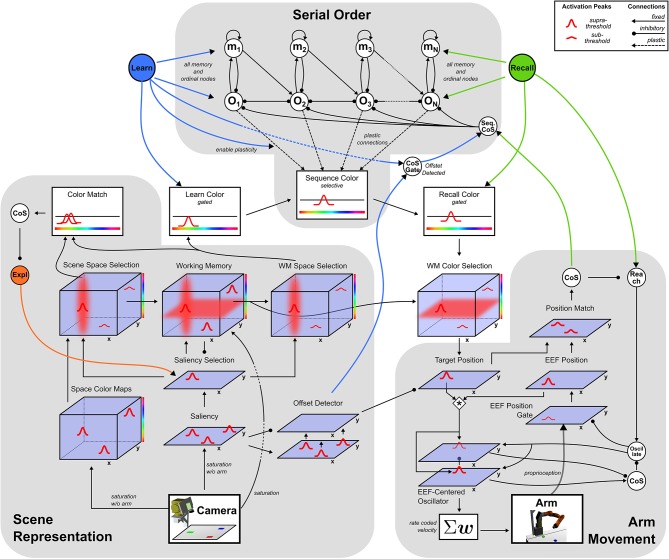
Sketch of the dynamic field network with its three sub-networks.

The neural dynamic architecture described here is a network of neural fields that are coupled to a camera and a robotic arm. These links enable online connection to a changing visual scene and online control of the arm. Three sub-networks (Figure 2) autonomously organize sequences of activation states to build visual representations, learn or perform serially ordered sequences, and generate object-oriented movements.

The perceptual sub-network, connected to the camera, creates a working memory representation of the visual scene through autonomous shifts of attention. A motor sub-network drives an oscillator generating velocity commands for the robotic arm. The cognitive sub-network represents ordinal positions in a sequence and may autonomously shift from one ordinal position to the next. The ordinal system may be used in two different manners, sequence learning and sequence recall, controlled by the activation of one of two different task nodes. These task nodes activate behaviors by boosting fields' resting levels and enabling fields to generate task relevant attractor states.

The following sections describe for each sub-network the states that drive behavior and the mechanism for how the system switches between those states. The last section addresses the integration of all three sub-networks for the two tasks *Learn* and *Recall*.

### 3.1. Perception: Scene Representation

The scene representation sub-network is based on Grieben et al. (2018) and creates three-dimensional (2D space and 1D color) working memory representations of objects in the visual scene captured by the camera. Each entry into the representation is created sequentially as the sub-network autonomously shifts attention across different objects in the scene.

The network's attention is modeled through peaks of activation in the two-dimensional *Saliency Selection* field that arise at salient locations in the scene. These locations are represented in the *Saliency* field which receives input directly from the camera. Based on their distinctive colors, the table and the robot's own arm are subtracted from the image in a pre-processing step. The saturation channel of the resulting HSV-image serves as input amplitude at each location.

Combined with a homogeneous boost of its resting level from the *Exploration* intention node, activation in the *Saliency* field is sufficient to create a single peak in the *Saliency Selection* field. Attentional shifts occur whenever the *Exploration* node deactivates and subsequently reactivates, causing a destabilization of the present peak in the *Selection* field followed by the emergence of a new peak at a new location. Previously unattended locations are more likely to be selected because inhibitory influence from the working memory gives them a competitive advantage.

The activation of the *Saliency Selection* field, *u*_sel_, is governed by the following neural dynamics:

(2) $$\begin{array}{l} \tau {\dot{u}}_{\text{sel}}(x,y)=-\;u_{\text{sel}}(x,y)+h_{\text{sel}}+w_{\text{exp}}\sigma (u_{\text{exp}})\\ \;+\;w_{\text{sal}}\sigma (u_{\text{sal}}(x,y))-w_{\text{mem}}\int \sigma (u_{\text{mem}}(x,y,c))dc\\ \;+\;\int \sigma (u_{\text{sel}}(x^{\prime },y^{\prime }))\omega_{\text{sel}}(x-x^{\prime },y-y^{\prime })dx^{\prime }dy^{\prime }, \end{array}$$

where *h*_sel_ describes the field's resting level, *u*_exp_ the homogeneous boost activation from the *Explore* intention node, *u*_sal_(*x, y*) the activation of the *Saliency* field, ∫ σ(*u*_mem_(*x, y, c*))*dc* the activation of the *Working Memory* projected onto the two spatial dimensions, *x* and *y*, and ω_sel_ the field's selective lateral interaction kernel. Each input, σ*u*_in_, to the field is weighted by a specific weight, *w*_in_. The same notation is used in all following equations and the concrete parameter values can be found in the Appendix.

The currently attended location achieves spatial feature binding: It is forwarded to the three 3D space-color fields, *Scene Space Selection, Working Memory*, and *WM Space Selection*, ensuring that the color features represented in those fields originate from the same location. The *Scene Space Selection* field combines sub-threshold activation from the *Space Color Maps* field, that represents color information in the scene, with spatial sub-threshold activation from the *Saliency Selection* field. Together, these inputs induce a single peak in three dimensions that represents the attended spatial location and the color perceived at that location.

That color is extracted and combined with spatial information from the *Saliency Selection* field to add a peak in the 3D-*Working Memory* field.^1^ The *Working Memory* field receives additional input directly from the camera image that includes the robot's arm. That input is proportional to the saturation channel from which the table color saturation has been subtracted. This mask, seen, for instance, in the Working Memory row of Figure 4, makes it possible to sustain peaks of activation anywhere where the camera picks up visual structure. Peaks representing objects can thus remain stable in working memory when they become occluded by the robot's arm, but are removed from working memory when they disappear from the scene at any other location.

The activation, *u*_mem_(*x, y, c*), of the *Working Memory* field is governed by the following dynamics:

(3) $$\begin{array}{l} \tau {\dot{u}}_{\text{mem}}(x,y,c)\\ =-\;u_{\text{mem}}(x,y,c)+h_{\text{mem}}+w_{\text{sel}}(x,y,c)\sigma (u_{\text{sel}}(x,y))\\ +\;w_{\text{nbk}}(x,y,c)\sigma (i_{\text{nbk}}(x,y))+w_{\text{ssl}}\int \sigma (u_{\text{ssl}}(x,y,c))dxdy\\ +\;\int \sigma (u_{\text{mem}}(x^{\prime },y^{\prime },c^{\prime }))\omega_{\text{mem}}(x-x^{\prime },y-y^{\prime },c-c^{\prime })dx^{\prime }dy^{\prime }dc^{\prime }, \end{array}$$

where *h*_mem_ describes the field's resting level, *u*_sel_(*x, y*) the activation from the *Saliency Selection* field, *i*_nbk_(*x, y*) the saturation channel from the camera image, ∫ σ(*u*_ssl_(*x, y, c*))*dxdy* the activation of the *Scene Space Selection* field projected onto the color dimension, *c*, and ω_mem_ the field's lateral interaction kernel.

Supra-threshold activation of the *Working Memory* field is forwarded to the *Memory Space Selection* field, which works analogously to the *Scene Space Selection* field and thus forms a single 3D activation peak, representing color and spatial location of the attended location in working memory.

Color information represented in the *Scene* and *Memory Space Selection* fields is forwarded to the *Color Match* field. That field forms a peak only when the input from the scene overlaps in location and color with one of the peaks in the memory field. A peak in the match field thus signals successful entry of an item into the *Working Memory* at the currently attended location. Supra-threshold activation in the match field projects onto the *CoS Explore* node, which in turn inhibits the *Explore* intention node. Deactivation of the *Explore* intention node removes the resting level boost from the *Saliency Selection* field inducing a reverse detection instability that propagates to the *Scene* and *Memory Space Selection* fields, the *Color Match* field and ultimately to the *CoS Explore* node. The newly created peak in the *Working Memory* field is sustained and the *Explore* intention node is released from inhibition enabling attentional selection of a new location.

#### 3.1.1. Offset Detector

The scene representation sub-network is capable of detecting sudden object movement with the help of a two-layer offset detector connected to the *Saliency* field. Both layers, *u*_dfa_ and *u*_dsl_, are two-dimensional fields over image space that are governed by the following dynamics with timescales, τ_dfa_ < τ_dsl_:

(4) $$\begin{array}{l} \tau_{\text{dfa}}{\dot{u}}_{\text{dfa}}(x,y)=-\;u_{\text{dfa}}(x,y)+h_{\text{det}}-w_{\text{sin}}\sigma (u_{\text{sal}}(x,y))\\ \;+w_{\text{dsl}}\sigma (u_{\text{dsl}}(x,y)),\\ \tau_{\text{dsl}}{\dot{u}}_{\text{dsl}}(x,y)=-u_{\text{dsl}}(x,y)+h_{\text{det}}+w_{\text{sex}}\sigma (u_{\text{sal}}(x,y)), \end{array}$$

where *h*_det_ describes the common resting level, and σ(*u*_sal_(*x, y*)) the thresholded activation of the *Saliency* field which excites the slower layer, *u*_dsl_, and inhibits the faster layer, *u*_dfa_. Because inhibitory input is stronger than excitatory input (*w*_sin_ > *w*_dsl_), static visual structure induces supra-threshold activation in the slow layer, *u*_dsl_, not in the fast layer, *u*_dsl_.

Once an object is removed from the scene, the inhibitory influence, *w*_sin_, vanishes faster than the excitatory influence from the slow layer, *u*_dsl_, leading to the formation of a peak in *u*_dfa_ that represents the detection of an object that moves away from the location of the peak.

### 3.2. Motor: Arm Movement

**Figure 3:**
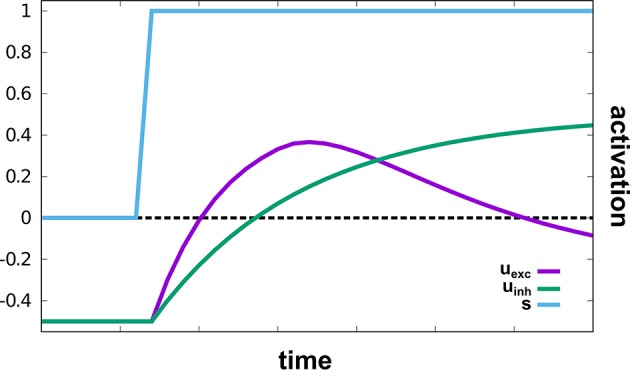
An active transient in *u*_exc_ generated by a two-layer oscillator in response to an input stimulus, *s*. The supra-threshold activation level leads to a bell-shaped velocity profile on read-out. This illustration is zero-dimensional while in the model, a two-dimensional field of identical oscillators is used.

The sub-network responsible for reaching movements, based on Schöner et al. (2019), autonomously drives an oscillator that creates velocity commands which move a robotic arm to a given target in two-dimensional space. A hierarchy of intention and CoS nodes governs the behavior: The *Reach* intention node activates the *Oscillate* intention node, which initiates an active transient (see Figure 3). The *Cos Oscillate* node is activated once the transient reaches a new steady state, while the *CoS Reach* is activated when the representations of target and end-effector position match. Thus, multiple active transients (oscillations) are generated until the arm reaches the represented target.

Target and end-effector (EEF) are both represented as peaks of activation in two-dimensional fields defined over image space, the *Target Position* and the *EEF Position* DNFs, respectively. Activation originating from working memory causes the creation of a peak in the *Target Position* field. Proprioceptive information from the current arm configuration is mapped through a forward kinematics into end-effector space and then transformed from rate to space code inducing a peak in a two-dimensional *EEF Position* field. Target and end-effector representations are cross-correlated with each other to create an end-effector centered representation of the target position. This representation is input into a two-layer field of neural oscillators, *u*_exc_ and *u*_inh_. The faster excitatory layer, *u*_*exc*_, generates an active transient illustrated in Figure 3: Its input first drives up excitation, which is then suppressed by inhibition from the slower inhibitory layer, *u*_inh_:

(5) $$\begin{array}{l} \tau_{\text{exc}}{\dot{u}}_{\text{exc}}(x,y)=-\;u_{\text{exc}}(x,y)+h_{\text{osc}}+w_{\text{cct}}\sigma (u_{\text{cct}}(x,y))\\ \;+w_{\text{osc}}\sigma (u_{\text{osc}})-w_{\text{inh}}\theta (u_{\text{inh}}(x,y))\\ \tau_{\text{inh}}{\dot{u}}_{\text{inh}}(x,y)=-u_{\text{inh}}(x,y)+h_{\text{osc}}+w_{\text{cct}}\sigma (u_{\text{cct}}(x,y))\\ \;+w_{\text{osc}}\sigma (u_{\text{osc}}), \end{array}$$

where τ_exc_ < τ_inh_ are the different relaxation times, *h*_osc_ the resting level, σ(*u*_cct_(*x, y*)) the end-effector-centered target representation, σ(*u*_osc_) the homogeneous resting level boost from the *Oscillate* intention node, and θ – a semi-linear threshold function.

The thresholded activation, θ(*u*_exc_(*x, y*)), is transformed into a rate coded Cartesian velocity vector, ***v***, using a set of feed-forward weights, ***w***_vel_(*x, y*):

(6) $$v(t)=\int \int w_{\text{vel}}(x,y)\theta (u_{\text{exc}}(x,y,t))dxdy$$

The weights, ***w***_vel_(*x, y*), describe a linear distance function in the end-effector centered representation of the target position. For different movement distances, (*x, y*), these weights are tuned such that the arm reaches the target position within a fixed movement time. The velocity vector, ***v***, is transformed into a joint velocity vector, $\overset{∙}{\lambda }$, using the pseudo-inverse of the arm's Jacobian, ***J***^+^, which depends on the current joint configuration ***λ***(*t*):

(7) $$\dot{\lambda }=J^{+}(\lambda (t))v(t)$$

For more details on the generated velocity profile see Schöner et al. (2019).

While the oscillator is going, its input is not updated, because the connection from proprioception to the *EEF-Position* field is gated by the *Oscillate* intention node. The *EEF-Position* field thus effectively represents the initial position of the hand. Termination of the transient is detected by the *CoS Oscillate* node, which receives excitatory activation from *u*_inh_ and inhibitory activation from *u*_exc_. Activation of *CoS Oscillate* inhibits the *Oscillate* intention node, which resets the oscillator, and releases the *EEF Position Gate* from inhibition so that the end-effector position is updated. When the target representation overlaps sufficiently with the updated EEF Position, a peak forms in the *Position Match* field and activates the *CoS Reach*, which terminates the reach.

### 3.3. Cognition: Serial Order

The serial order sub-network, based on Sandamirskaya and Schöner (2010), allows for the autonomous storage and recall of a sequence of activation patterns. Each activation pattern is represented through learned inhomogeneous connections between an *ordinal* node and a feature field, here the one-dimensional *Sequence Color* field. Supra-threshold activation in a particular ordinal node thus induces a peak in the *Sequence Color* field that represents the color associated with that particular stage in the sequence.

The sub-network consisting of ordinal nodes, memory nodes and a single CoS node enforces the sequential activation of ordinal nodes in a fixed order:

(8) $$\begin{array}{l} \;\tau {\dot{o}}_{\text{i}}=\;-\;o_{i}+h+w_{o_{i},o_{i}}\sigma (o_{i})-w_{o_{i},o_{j}}\sum_{j\neq i}\sigma (o_{j})+w_{{\text{m}}_{\text{i}-\text{1}},{\text{o}}_{\text{i}}}\sigma (m_{i-1})\\ \;-\;w_{{\text{m}}_{\text{i}},{\text{o}}_{\text{i}}}\sigma (m_{i})-w_{\text{CoS}}\sigma (u_{\text{CoS}})+w_{{\text{oh}}_{\text{+}}}\sigma (u_{\text{lrn}})\\ \;+\;w_{{\text{oh}}_{\text{+}}}\sigma (u_{\text{rcl}})\\ \tau {\dot{m}}_{i}=\;-\;m_{i}+h+w_{m_{i},m_{i}}\sigma (m_{i})+w_{{\text{o}}_{\text{i}},{\text{m}}_{\text{i}}}\sigma (o_{i})+w_{{\text{mh}}_{\text{+}}}\sigma (u_{\text{lrn}})\\ \;+w_{{\text{mh}}_{\text{+}}}\sigma (u_{\text{rcl}}). \end{array}$$

An active ordinal node, *o*_*i*_, representing the *i*th position in the sequence, inhibits all other ordinal nodes, *o*_*j*_, and activates its own self-sustained memory node, *m*_*i*_. The memory node pre-activates the next ordinal node, *o*_*i*+1_, through an excitatory connection and inhibits its own ordinal node, *o*_*i*_, to prevent it from becoming reactivated after completion of the stage. While activated, an ordinal node's self excitation, *w*_*o*_*i*_,*o*_*i*__, is sufficient to overcome inhibition from its memory node, *w*_m_i_,o_i__. An ordinal node remains active until the CoS node, *u*_CoS_, is activated and destabilizes all ordinal nodes, which, in turn, removes input from the CoS node that deactivates. The self-sustained memory nodes are unaffected, so that upon release from inhibition by the CoS, the pre-activated ordinal node of the next element in the sequence is activated. Recurring activation and deactivation of the CoS node thus creates a sequence of autonomous transitions between sequence elements in the order of ascending *i*. Ordinal and memory nodes can become activated only in the presence of an excitatory boost, *w*_h_+__, from one of the task nodes, *Learn* (*u*_lrn_) or, *Recall* (*u*_rcl_). Deactivation of an active task node leads to deactivation of all memory and ordinal nodes, effectively resetting the entire system.

Connection weights, *w*_*o*_*i*_,*u*_col__, between the active ordinal node, *o*_*i*_, and the active region in the *Sequence Color* field, *u*_col_, are strengthened according to a dynamic version of the Hebbian learning rule:

(9) $$\tau {\dot{w}}_{o_{i},u_{\text{col}}}(c)=\eta \sigma (u_{\text{lrn}})\sigma (o_{i})(\sigma (u_{\text{col}}(c))-w_{o_{i},u_{\text{col}}}(c)),$$

where η describes the learning rate and *u*_lrn_ the activation of the *Learn* task node that gates the learning process.

Before learning, peaks in the *Sequence Color* field arise when a color attended in the Working Memory Selection field is input through the gate field, *Learn color*, *u*_lcol_:

(10) $$\begin{array}{l} \tau {\dot{u}}_{\text{col}}(c)=-\;u_{\text{col}}(c)+h+\int \sigma (u_{\text{col}}(c^{\prime }))\omega_{\text{col}}(c-c^{\prime })dc^{\prime }\\ \;+w_{\text{lcol}}\sigma (u_{\text{lcol}}(c))+\sum_{i}w_{o_{i},u_{\text{col}}}(c)\sigma (o_{i}), \end{array}$$

After learning, peaks in the Sequence color field may arise from previously learned connections, *w*_*o*_*i*_,*u*_col__(*c*), of an ordinal node, *o*_*i*_. The selective kernel, ω_col_, ensures that only a single color is represented at all times.

### 3.4. Task Integration: Learn and Recall

The full network may operate in two different regimes: In the learning regime, a sequence of colors is presented to the system and learned. In the recall regime, a learned sequence of colors is reproduced by pointing at colored objects in a specific order. Each regime is evoked by the activation of its corresponding task node, *Learn* and *Recall*, which alter the resting level of certain sub-sets of fields.

Both task nodes boost the resting level of all ordinal and memory nodes to allow supra-threshold activation. When task nodes are deactivated, the removal of the corresponding boost causes activation of all self-sustained nodes to decay, effectively resetting the system. This happens, for instance, at the end of the sequence due to activation of the sequence's condition of satisfaction.

The Learn node acts as a gate between the Scene Representation and the Serial Order sub-networks. By boosting the *Learn Color* field, the Learn node enables that field to form supra-threshold peaks. At which color such a peak is erected is controlled by input from the *Memory Space Selection* field that represents the color at the currently attended location. That color is then imprinted in the connections to the currently active ordinal node through the learning dynamics (Equation 9). The *Learn* node pre-activates the *Offset Detected* node, which connects to the *Sequence CoS*. Thus, whenever a single object is presented in the learning regime, its color is associated with the currently active ordinal node and its removal from the scene causes a transition in which the active ordinal node is replaced by the next ordinal node.

The Recall node is a gate between the sequence generation and the arm movement sub-networks. It boosts the *Recall Color* gating field so that the color represented in the *Sequence Color* field is passed on to the three-dimensional *Memory Color Selection field*. If an object in working memory overlaps with that color, a peak forms in the *Memory Color Selection* field. The peak's spatial position is forwarded to the *Target Position* field of the Arm Movement sub-network, which initiates a reaching movement. Once a reach has been successfully performed, the *Reach* CoS is activated, which triggers the *Sequence CoS*, causing the transition to the next ordinal node. In the recall regime, the arm will thus move autonomously to colored objects in the learned order, as long as appropriately colored objects are visible in the scene.

## 4. Results

In this section we show how activation within the network unfolds in time during the learn and recall tasks. We visualize relevant activation fields to illustrate how the network's autonomy enables it to cope with variable timing during learning and with changes of the scene during recall.

The network is effectively a large dynamical system. We solved it numerically on digital computers, and that numerical solution was the only form in which algorithms intervened in the system. The numerical implementation of the model made use of Cedar (Lomp et al., 2016), an open source framework in which DFT models can be graphically assembled and interactively tuned. Cedar can be used to simulate robotic behavior, which was done for the results illustrated in this paper. The visual scene, camera, and robot arm were simulated using Webots (Michel, 2004) that can be coupled into *Cedar*. The same *Cedar* code can also link to real sensors and robots. We did this, driving the model from a real camera and manipulating the visual scene by placing colored objects on a white table top. We also controlled a lightweight KUKA arm from the same *Cedar* code to verify its capacity to act out the planned movements. These informal robotic experiments are not further documented in this paper.

### 4.1. Scene Representation: Autonomous Build-up of Visual Working Memory

**Figure 4:**
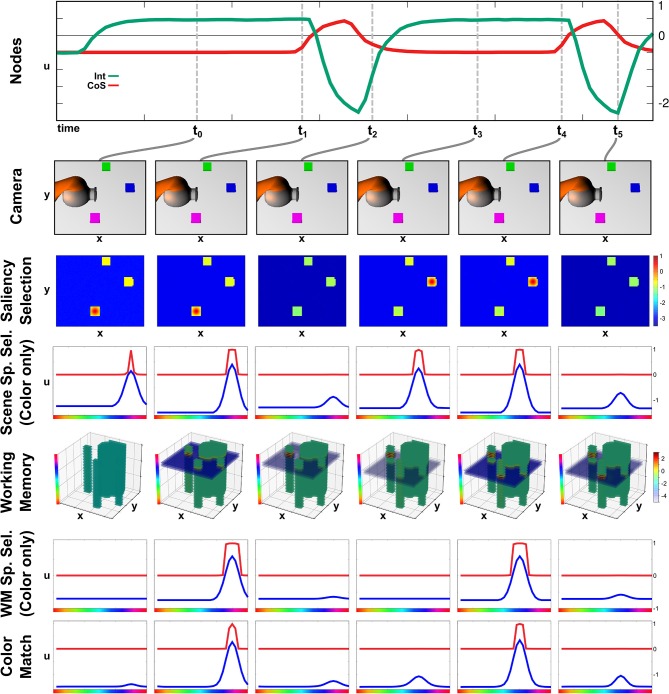
Time course of building a scene memory.

The build-up of the scene working memory is an ongoing process that provides visual information to the network irrespective of the currently active task node. In Figure 4 we show activation snapshots of different points in time during working memory build-up in an exemplary scene containing three objects and the arm's end-effector.

At point *t*_0_, the *Exploration* intention node provides a homogeneous boost to the *Saliency Selection* field leading to an activation peak at the location of the purple object. This causes the emergence of a three-dimensional peak in the *Scene Selection* field, of which the color dimension is shown in the third row. The *Working Memory* field contains no supra-threshold activation yet but, at the locations of the non-background objects, the resting level is increased across the whole color dimension.

Once the peak in the *Scene Selection* field has fully emerged at *t*_1_, its color component is forwarded as a slice toward the *Working Memory*, where it overlaps with the tube originating from the *Saliency Selection* field and forms a three-dimensional peak. Subsequently a peak also forms in the *Memory Spatial Selection* field, which shares the same color as the peak in the *Scene Space Selection* causing an overlap in the *Color Match* field.

The peak forming in the *Color Match* field activates the *CoS Explore* node, which inhibits the *Explore* intention node. Thus the resting level boost is removed from the *Saliency Selection* field, which subsequently falls down to sub-threshold activation at point *t*_2_. Only the self-sustained peak in the *Working Memory* field remains.

The absence of a peak in the *Color Match* field causes the CoS node to fall below threshold again, bringing the sub-network to its initial state. The following activation of the *Explore* intention node, depicted from *t*_3_ until *t*_5_, follows the same temporal activation pattern as the previous one with different feature values for spatial location and color. The spatial location in the *Saliency Selection* field differs due to the inhibitory influence from the *Working Memory* field. See Supplementary Video 3 for a different example of autonomous build-up of visual working memory in continuous time.

### 4.2. Learning Demonstration

**Figure 5:**
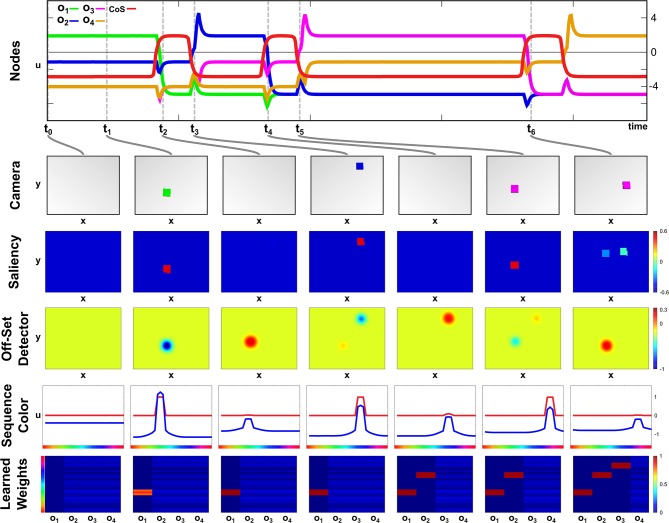
Time course of learning a three element sequence with varying presentation time.

A particular color sequence is taught to the network in its learning regime by presenting objects of a certain color one after another. In Figure 5 activation snapshots of some points in time during an exemplary learning episode are shown. The top row depicts the temporal evolution of activation of the ordinal nodes and the *Sequence CoS* node, while each snapshot column shows the camera image, the activation of the *Saliency* field, activation of the fast layer of the *Offset Detector*, activation of the *Sequence Color* field, and the weight values, *w*_*o*_*i*_,*u*_col__, for each ordinal node at one particular point in time.

In the initial phase of the learning at point *t*_0_ no objects are in the scene, but the *Learning* task node has been activated leading to supra-threshold activation in the first ordinal node. All other ordinal nodes are below threshold activity with a slight advantage for *o*_2_, which already receives an excitatory bias through the active memory node, *m*_1_.

At *t*_1_, a green object is inserted into the scene, which forms a peak in the *Saliency* field leading to a localized inhibition in the fast *OffSet Detector* field. It is also committed to working memory and leads to the emergence of a peak in the *Sequence Color* field encoding the green color. Due to present supra-threshold activation in the *Sequence Color* field and the ordinal node *o*_1_, the Hebbian learning rule strengthens weights between the ordinal node and the green color feature values.

The object is removed from the scene at *t*_2_, which destabilizes the peak in the *Saliency* field removing the inhibition from the fast layer of the *Offset Detector*. The slow layer (not depicted) still carries supra-threshold activation, exciting the fast layer leading to the formation of a peak, which will subsequently activate the *Sequence CoS* node inhibiting all ordinal nodes. This deactivates *o*_1_ and causes the color peak in the *Sequence Color* field to vanish as it is no longer supported by either learned connections nor color input from the scene. The missing input in the scene will also ultimately lead to a decay of activation in the slow *Offset Detector* layer and subsequently cause a reverse-detection instability in the fast layer and the *Sequence CoS* node.

The deactivation of the *Sequence CoS* node is followed by an activation of the next ordinal node *o*_2_ at *t*_3_. Between *t*_2_ and *t*_3_ a blue object has been added to the scene, whose color is then connected to the freshly activated ordinal node via the Hebbian learning rule. Removal of the object at *t*_4_ triggers the *Offset Detector* and the CoS node enabling the activation of the next ordinal node *o*_3_ at *t*_5_. The presented purple object is kept in the scene for a longer time span than the green or blue one, which does not influence the learning as the transition to the next sequence element at *t*_6_ is based on the removal event rather than timing.

### 4.3. Recall Demonstration

**Figure 6:**
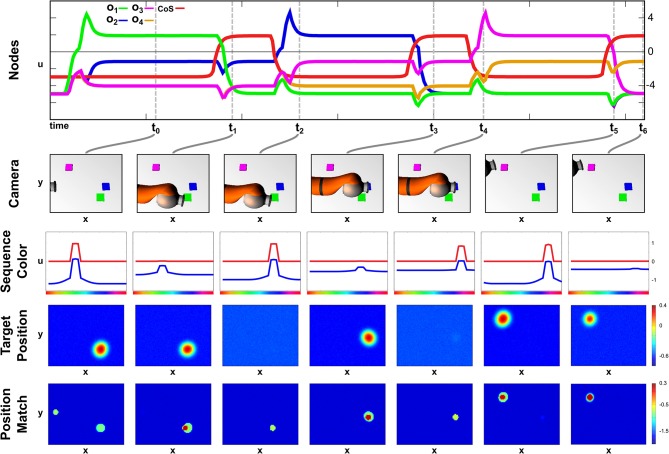
Time course of recalling a three element sequence through pointing at colored objects.

We demonstrate successful sequence recall through a pointing task, where the network moves the arm to an object in the scene matching the color of the current sequence element. Only a successful reach toward that object allows a progress to the next sequence element. An exemplary recall of three sequence elements is depicted in Figure 6, which demonstrates the temporal evolution of the activation of ordinal nodes as well as the field activity of the *Sequence Color*, the *Target Position*, and the *Position Match* field at discrete points during the sequence recall.

At point *t*_0_, the *Recall* task node has been activated, which lead to the activation of the first ordinal node and the emergence of a peak in the *Sequence Color* field at the green location due to the learned connections, *w*_*o*_1_,*u*_col__. The color information converges with the content of the *Working Memory* field in the *Memory Color Selection* field to form a three dimensional peak specifying position and color. Positional information is projected to the *Target Position* field of the Arm Movement sub-network, where it is forwarded to the movement generating oscillator and the *Position Match* field, which compares the current end effector position (center/left) with the current target position (bottom/right).

Due to a successful arm movement both positions match at point *t*_1_, which is represented through a peak in the *Position Match* field that activates the *Sequence CoS* deactivating the current ordinal node. The CoS node itself falls below threshold activity as soon as the peak in the *Position Match* field destabilizes through a missing target representation that vanished through insufficient color input from the *Sequence Color* field.

The missing inhibition from the CoS causes an activation of the next ordinal node *o*_2_, which is associated with blue color. At *t*_2_ however the blue peak has emerged in the *Sequence Color* field, but the target position has not yet been extracted from working memory. The column of point *t*_3_ depicts the end of the movement, where the overlap of end effector and target cause a peak that triggers the *Sequence CoS*. In this particular configuration the match representation is only possible due to the self-sustaining working memory representation that shields the blue object representation from the occlusion through the arm.

The movement toward the purple object depicted from *t*_4_ until *t*_6_ follows an analog activation pattern in which the ordinal node causes the formation of a purple peak in the *Sequence Color* field, which causes an extraction of the target position, leading to movement that terminates due to an represented match of positions. The movement times of all three movements are roughly the same despite their differences in distance, which results from the movement oscillator that enforces the same movement timing for all movements. See Supplementary Video 4 for another sequence recall demonstration showing the activation development of selected fields in continuous time.

#### 4.3.1. Recall With a Moving Object

**Figure 7:**
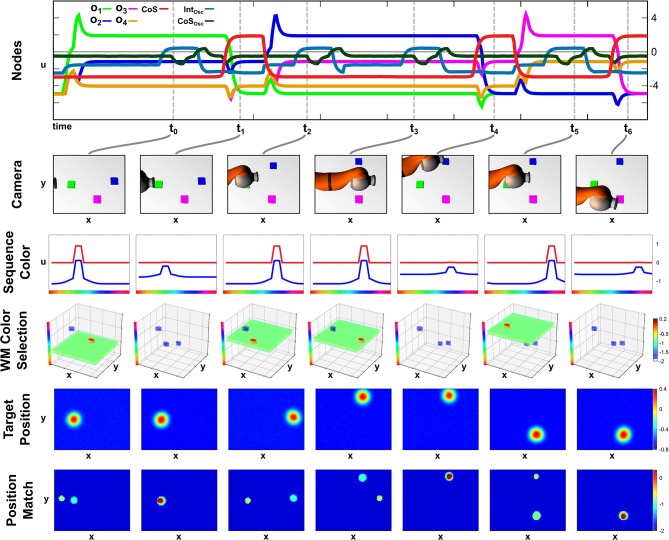
Online updating of the movement during sequence recall.

The autonomy of all three parts of the field network makes the execution of the recall task robust against unforeseen changes in the scene. We demonstrate this in an exemplary recall episode, where one of the objects in the scene is moved while its color corresponds to the active sequence element. The episode is depicted in Figure 7, which shows activation snapshots analog to Figure 6. Additionally activation of the Intention and CoS node driving the two-layer oscillator are shown as well as snapshots of the *Memory Color Selection* field.

In this episode, build-up of the scene memory starts simultaneously with activation of the recall task, which causes a delay between the activation of the first ordinal node and the first movement as the green object, which is the first sequence element, is the second object committed to memory. This can be observed at *t*_0_ in the *Memory Color Selection* field, where the green object forms a peak as it overlaps with the green color slice specified by the sequence color, while the purple object is present as a sub-threshold activation blob, and the blue object is entirely absent. As the first movement is finished at *t*_1_ all three objects are present in working memory as sub-threshold activation blobs.

Thus at *t*_2_, the second movement starts closely after the activation of the second ordinal node with the blue object as the target on the right side of the camera image. While the arm is moving the object is moved to the center/top position of the image, which results in a non-match between arm and target at the end of the movement, which can be seen at *t*_3_. Here working memory has updated the position of the blue object, which leads to an extraction of a different target position that does not match with the current position of the end effector. Only at *t*_4_ after a second movement was generated, the blue object and the end effector match, which concludes the recall of the second element of the sequence.

The last movement toward the purple object is then conducted without any further perturbations and terminates after a single movement at *t*_6_.

#### 4.3.2. Recall With a Missing Object

**Figure 8:**
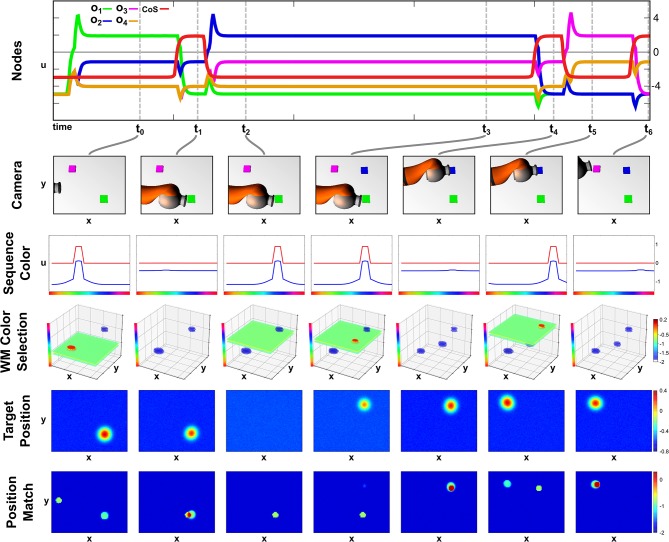
Recall with a delayed second object.

In this second recall episode demonstrating the robustness of the field network we start the recall in a scene that lacks the second object of the sequence. In Figure 8, activation snapshots of the same sub-set of fields used in the previous perturbation episode are shown.

At points *t*_0_ and *t*_1_, the network's activation develops analog to the previous two recall examples with a color slice used to extract the target position and the position match to determine the successful termination of the movement. However as the second ordinal node activates at *t*_2_ no blue object is present in the scene, thus no sub-threshold activation blob overlaps with the blue color slice in the *Memory Color Selection* field and no peak forms.

At point *t*_3_, the blue object is added to the scene, which is committed to memory and afterwards extracted as a valid target position. The movement than concludes at *t*_4_ with the arm occluding the purple object, which is kept in working memory due to the self-sustaining kernel. The working memory information is then used in *t*_5_, when the third ordinal node specifies purple as the next sequence color. Thus the sequence ends at *t*_6_ with no further perturbations.

## 5. Discussion

We have presented a network of dynamic neural fields that integrates the complete pathway from the sensor surface (vision) to representations of higher cognition (serial order) and to the motor system (pointing). The network architecture enables a robotic agent to autonomously learn a sequence of colors from demonstration and then to act according to the defined serial order on a scene. Both during learning and while acting out the sequence, the transitions between elements of the sequence are detected without the need for an external control signal (The switch between learning and recall mode is not autonomous, however, reflecting a similar need for task instructions when a human operator performs such a task).

In each of the three sub-networks responsible for scene representation, the representation of serial order, and movement generation, sequential transitions between neural activation states are brought about through the mechanism of the condition of satisfaction. Thus, visual attention shifts only once a currently attended item has been committed to working memory. A transition to the next element in the serial order occurs only once the robot has successfully acted on the current element. And an arm movement terminates only once the desired movement target has been reached. The mechanism of the condition of satisfaction thus reconciles the capacity to autonomously act according to learned or structurally determined plans with the capacity to be responsive to sensory or internal information about the achievement of goals.

### 5.1. What the Scenario Stands for

The scenario was simple, but meant to demonstrate the fundamental components of any neurally grounded autonomous robot.

1. A representation of the visual surround is the basis for any intelligent action directed at the world. It is also the basis for sharing an environment with a human user. We humans are particularly tuned to building scene representations which form the basis of much of our visual cognition (Henderson and Hollingworth, 1999). Scene representations need to include scene memory to deal with occlusions (e.g., by the agent's own body or body parts of a collaborating human user) and with a limited viewing range. Scene representations must also be open to updating, however, when the scene changes over time. Attentional selection is the key process that provides an interface between the scene and any action plan. So, while we stripped the system down to the bare essentials, the core processes of scene representation were covered.
2. Directing action to objects in the world requires transforming attentionally selected scene information into a coordinate frame anchored in the initial position of the actuator. In that representation, motor plans can be framed as movement parameters (Erlhagen and Schöner, 2002) that characterize the movement as a whole. Movements must be initiatiated and terminated, and time courses of motor commands must be generated that take the effector to the target. In dynamic environments, such as when a human user interferes with objects, the movement parameters must be open to online updating. If movements still fail to reach the target, correction movements must be generated. Even in our extremely limited implementation, these core processes of movement generation were covered. Control issues, which are not trivial in human movement but are well-understood in robotics, were neglected.
3. The cognition of goal-directed action was simplified to serial order. Serial order is a cognitive construct in that it abstracts from the contents (what is serially ordered) and from time (when is each item addressed). Based on these abstractions, a broad set of actions can be conceived of as serially ordered processing steps. For instance, assembling a piece of IKEA furniture could be described this way. Unlike many classical, disembodied cognitive tasks, real action sequences require the capacity to deal with variable and perhaps unpredictable amounts of time needed to achieve each processing step. Learning the—a priori—arbitrary contents of a serially ordered sequence makes this scenario quite powerful. It goes beyond, for instance, a mere capacity to imitate or emulate behavior, which would lead to the reproduction of the same movements or effects without generalization to new conditions. It also goes beyond the generation of sequences of behaviors that would be triggered by environmental conditions according to a fixed organizational scheme encoded in a behavior-based robotic architecture (Elements of fixed sequencing are contained in the present system such as when attentional selection always precedes pointing).

### 5.2. Scaling Beyond the Simplified Scenario

1. The scene representation system was built on a single, trivial color feature. We have previously explored and demonstrated elsewhere how a neural dynamic system of the same kind can deal with multiple feature dimensions (e.g., Chapter 8 of Schöner et al., 2016 or Grieben et al., 2018). This entails a feature binding problem that can be solved through a shared spatial dimension across multiple low-dimensional feature/space fields. Binding occurs by attending selectively to a spatial location and transmitting feature information separately in the different feature/space fields. In this account, such binding through space is ultimately the reason why objects need to be attended sequentially. Active gaze shifts would be another extension, the basis for which has been outline in previous work (e.g., Wilimzig et al., 2006). A further generalization would be to extend the scene representation into a semantic map in which objects are also classified and their class labels are stored. There is, of course, a plethora of neurally based feedforward classification system (reviewed in the section 1), and their outputs could be treated as such labels. Such modules may need improvement in terms of object segmentation and pose estimation (as discussed in Lomp et al., 2017), which clearly requires attentional processes. Visual search for such labels is a challenge that may need some research attention.
2. The motor domain is, of course, a much richer domain than we were able to demonstrate. There are myriad problems to be solved such as dealing with many degrees of freedom, dealing with obstacles, dealing with compliant actuators, controlling impedance, grasp planning and control, manipulating objects dynamically, and many more. For many of these, technical solutions are available or are being actively researched. Neurally grounded process accounts have not been developed as strongly as one would hope, however. The fundamental difference between human motor control and the control of current robot arms limits the extent to which approaches inspired by modern control theory and the theory of optimal control carry over to neurally inspired robotics (but see Driess et al., 2018).
3. We have looked at the learning and recall of a single sequence. To learn multiple sequences, additional processing substrate must be introduced that represents activation of such learned sequences as well as selection of a neural population when a new sequence is added to the sequence memory. In principle, an approach inspired by Adaptive Resonance Theory (Carpenter and Grossberg, 2017) may achieve that. We have outlined such an approach in related work on contingency learning (Tekülve and Schöner, 2019), but important questions remain open such as how to align sequences of different lengths. The position encoding of serial order in the ordinal nodes makes it possible, however, to represent sequences that entail the same elements in different serial positions (Sandamirskaya and Schöner, 2010).

The sliver of cognition we have captured may be part of communication, showing each other what to do. If perception was better (e.g., recognizing events and perceiving relationships between actuators and objects), and if action was richer (e.g., the ability to use tools and manipulate objects), then the modeled interface would already make the robot quite useful. It would enable a robot to learn the solution of problems from a human user, as long as the perception system extracts the conceptual structure of the demonstrated action. A big extension would be the capacity of the system to solve problems by itself, devising the sequences of actions required to achieve a goal. This would require neural processes in new domains such as exploration, outcome representations, perhaps value systems. There is a growing literature on such models (Mnih et al., 2015), but their import for robotic learning is an open research problem.

### 5.3. Related Work

A number of groups have addressed object-directed action and the requisite perception in a similar neural-dynamic framework (Fard et al., 2015; Strauss et al., 2015; Tan et al., 2016). Serial order and the specific neural mechanism for sequencing neural activation patterns were not yet part of these efforts, which otherwise overlap with ours. A number of neural dynamic models of serial order or sequencing have been proposed (e.g., Deco and Rolls, 2005), but not been brought into robotic problems. One reason may be the lack of a control structure comparable to our condition of satisfaction, so that the sequences unfold in neural dynamics at a given rhythm that is not synchronized with perceptual events. Such systems would not remain tied to the actual performance of a sequence in the world.

Related attempts to model in neural terms the entire chain from perception to action have been made for robotic vehicles. For instance, Alexander and Sporns (2002) enabled a vehicle to learn from reward a task directed at objects that a robot vehicle was able to pick up. pick up. Gurney et al. (2004) realized a neurally inspired system the organized the organism (This paper is useful also for its careful discussion of different levels of descriptions for neurally inspired approaches to robotics). Both systems are conceptually in the fold of behavior-based robotics, in that the sequences of actions emerge from a neural architecture, modulated by adaptation. To our knowledge, systems of that kind have not yet been shown to be able to form serial order memories and acquire scene representations.

A different style of neural robotic model for cognition is SPAUN (Eliasmith et al., 2012). This is an approach based on the Neural Engineering Framework (Eliasmith, 2005), which is able to implement any neural dynamic model in a spiking neural network. Thus, models based on DFT may, in principle, be implemented within this framework. On the other hand, SPAUN has also been turned to approaches to cognition that may not be compatible with the principles of DFT, in particular, the Vector Symbolic Architecture (VSA) framework that goes back to Smolensky, Kanerva, Plate, and Gayler (see Levy and Gayler, 2008 for review). In VSA, concepts are mapped onto high-dimensional vectors, that enable processing these concepts in the manner of symbol manipulation. If this approach is entirely free of non-neural algorithmic steps is not clear to us.

## 6. Conclusion

We have shown, in a minimal scenario, how sequences of attentional shifts, of movements, and of serially ordered actions can be autonomously generated in a neural dynamic framework that is free of any non-neural algorithmic control. The continuous or intermittent coupling to sensory and motor systems is made possible by creating neural attractor states. Inducing instabilities in a controlled manner enables the system to make sequential transitions between such states. As a result, the neural dynamic robot demonstrates a minimal form of cognition, learning and acting out serially ordered actions. Much work remains to be done to scale such systems to the real world.

## Data Availability Statement

All relevant data to reproduce the presented dynamic field network with our open-source software cedar (cedar.ini.rub.de) is contained within the manuscript. For technical questions regarding the software please contact the authors.

## Author Contributions

JT and AF performed the work, and contributed to the writing of the manuscript. YS and GS conceived of the project, provided research supervision, and contributed to the writing of the manuscript.

### Conflict of Interest

The authors declare that the research was conducted in the absence of any commercial or financial relationships that could be construed as a potential conflict of interest.

## Footnotes

^1^Spatial information is taken from the *Saliency Selection* field rather than directly from the *Scene Space Selection* field to allow for possible coordinate transforms between image and memory space. In the present scenario, the camera is not moved so that there is no need for a coordinate transform.

## Appendix

### Network Parameters

The following tables list the field parameters of the different sub-networks. Lateral kernels are constructed according to the following equation: $\omega \left(\text{a},\sigma ,c_{\text{inh}}\right)=c_{\text{inh}}+\underset{i}{\sum }a_{i}\frac{1}{\sqrt{2\pi {\sigma_{i}}^{2}}}\exp\left(\frac{-x^{2}}{2{\sigma_{i}}^{2}}\right)$. The learning rate used in Equation (9) is η = 0.05.

**Table A1:** Parameter values of the scene representation sub-network.

**Name**		**Resting level**	**Lateral Kernel**	**Inputs**
**SCENE REPRESENTATION**				
Saliency	*u*_sal_	*h*_sal_ = −0.5	ω_sal_: *a*_1_ = 1, σ_1_ = 3, *c*_inh_ = −0.0001	*w*_img_ = 1
Saliency selection	*u*_sel_	*h*_sel_ = −2.5	ω_sel_: *a*_1_ = 3, σ_1_ = 2, *c*_inh_ = −0.08	*w*_sal_ = 2.2, *w*_exp_ = 2.0, *w*_mem_ = −0.1
Space color maps	*u*_scm_	*h*_scm_ = −0.5	ω_scm_: *c*_inh_ = −0.0001	*w*_img_ = 1
Scene space selection	*u*_ssl_	*h*_ssl_ = −1.5	ω_ssl_:*c*_inh_ = −0.0001	*w*_scm_ = 1, *w*_sel_ = 1
Working memory	*u*_mem_	*h*_mem_ = −5	ω_mem_: *a*_1_ = 5, σ_1_ = 2, *a*_2_ = −2.5, σ_2_ = 6, *c*_inh_ = −0.0001	*w*_sel_ = 1, *w*_nbk_ = 3.5, *w*_ssl_ = 1
WM space selection	*u*_wsl_	*h*_wsl_ = −1.5	ω_wsl_: *c*_inh_ = −0.0001	*w*_mem_ = 1, *w*_sel_ = 1
Color match	*u*_cm_	*h*_cm_ = −1.5	ω_cm_: *c*_inh_ = −0.0001	*w*_ssl_ = 1, *w*_wsl_ = 1
Explore int	*u*_exp_	*h*_sel_ = −0.5	—	*w*_ecs_ = −3
Explore CoS	*u*_ecs_	*h*_ecs_ = −0.5	—	*w*_cm_ = 1
Off-set detector fast	*u*_dfa_	*h*_dfa_ = −0.2	—	*w*_sin_ = −1, *w*_dsl_ = 0.5
Off-set detector slow	*u*_dsl_	*h*_dsl_ = −0.2	—	*w*_sex_ = 1

**Table A2:** Parameter values of the arm movement sub-network.

**Name**		**Resting level**	**Lateral Kernel**	**Inputs**
**ARM MOVEMENT**				
Target position	*u*_tp_	*h*_tp_ = −0.5	ω_tp_: *c*_inh_ = −0.001	*w*_wcl_ = 1, *w*_dfa_ = −2
EEF position	*u*_ep_	*h*_ep_ = −0.5	ω_ep_: *a* = 25, σ = 2, *c*_inh_ = −0.3	*w*_epg_ = 10
EEF position gate	*u*_epg_	*h*_epg_ = −0.5	ω_epg_: *c*_inh_ = −0.001	*w*_ppc_ = 1, *w*_oin_ = −2
Position match	*u*_pm_	*h*_pm_ = −1.5	—	*w*_ep_ = 1, *w*_tp_ = 1
Oscillator fast	*u*_ofa_	*h*_ofa_ = −2.5	ω_ofa_: *c*_inh_ = −0.001	*w*_oin_ = 2, *w*_efc_ = 1, *w*_osl_ = −4
Oscillator slow	*u*_osl_	*h*_osl_ = −2.5	ω_osl_: *c*_inh_ = −0.001	*w*_oin_ = 2, *w*_efc_ = 1
Oscillate intention	*u*_oin_	*h*_oin_ = −0.5	—	*w*_rea_ = 1, *w*_ocs_ = −2
Oscillate CoS	*u*_ocs_	*h*_ocs_ = −0.5	—	*w*_ofa_ = −1, *w*_osl_ = 1
Reach intention	*u*_rea_	*h*_rea_ = −0.5	—	*w*_rcl_ = 1, *w*_rcs_ = −2
Reach CoS	*u*_rcs_	*h*_rcs_ = −0.5	—	*w*_pm_ = 1

**Table A3:** Parameter values of the serial order sub-network.

**Name**		**Resting level**	**Lateral Kernel**	**Inputs**
**SERIAL ORDER**				
Ordinal node	*o*_*i*_	*h*_o_ = −5	*w*_*o*_*i*_,*o*_*i*__ = 4.8	*w*_*o*_*i*_,*o*_*j*__ = 2, *w*_*m*_*i*−1_,*o*_*i*__ = 2.9, *w*_*m*_*i*_,*o*_*i*__ = 3.8, *w*_CoS_ = 2, *w*_*o*_*h*__+__ = 3
Memory node	*m*_i_	*h*_m_ = −5	*w*_*m*_*i*_,*m*_*i*__ = 5	*w*_*o*_*i*_,*m*_*i*__ = 2.6, *w*_*m*_*h*__+__ = 3
Sequence CoS	*u*_CoS_	*h*_CoS_ = −0.5	—	*w*_rcs_ = 1, *w*_dfa_ = 1
Sequence color	*u*_col_	*h*_col_ = −0.3	ω_col_: *a* = 1, σ = 3, *c*_inh_ = −0.3	*w*_lcol_ = 1, *w*_*o*_*i*_,*u*_col__∈[0, 1]

**Table A4:** Parameter values of task related fields of the dynamic field network.

**Name**		**Resting level**	**Lateral Kernel**	**Inputs**
**LEARN AND RECALL**				
Learn node	*u*_lrn_	*h*_lrn_ = −0.5	—	*w*_manual_ = 1
Recall node	*u*_rcl_	*h*_rcl_ = −0.5	—	*w*_manual_ = 1
Learn color	*u*_lcol_	*h*_lcol_ = −1.5	—	*w*_lrn_ = 1, *w*_wsl_ = 1
Recall color	*u*_rcol_	*h*_rcol_ = −1.5	—	*w*_col_ = 1, *w*_rcl_ = 1
WM color selection	*u*_wcl_	*h*_wcl_ = −1.5	ω_wcl_: *c*_inh_ = −0.0001	*w*_rcol_ = 1, *w*_mem_ = 1
